# Prevalence and associated factors of sexual violence experienced by housemaids in Ethiopia: a systematic review and meta-analysis

**DOI:** 10.1186/s12978-022-01470-2

**Published:** 2022-07-19

**Authors:** Birye Dessalegn Mekonnen, Zemene Habtu Lakew, Endalkachew Belayneh Melese

**Affiliations:** 1 Department of Nursing, Teda Health Science College, P.O. Box 790, Gondar, Ethiopia; 2 School of Medicine, College of Medicine and Health Sciences, University of Gondar, P.O. Box 196, Gondar, Ethiopia

**Keywords:** Prevalence, Housemaid, Sexual violence, Systematic review, Ethiopia

## Abstract

**Background:**

Housemaids often experience different types of sexual violence by different perpetrators. Sexual violence against housemaids remains usually concealed as victims cannot report such offenses. Except for fragmented studies with varying reports, there is no national prevalence studies conducted on sexual violence among housemaids in Ethiopia. Thus, this systematic review and meta-analysis aimed to determine the pooled prevalence and associated factors of sexual violence amongst housemaids in Ethiopia.

**Methods:**

A systematic search of PubMed, Embase, Science Direct, HINARI, Scopus, Cochrane Library, and Google Scholar was conducted using relevant search terms. Data were extracted using the Joanna Briggs Institute (JBI) tool. The quality of all selected articles was evaluated using JBI critical appraisal checklist. Data analysis was performed using STATA Version 14 statistical software. Egger’s test and funnel plot were used to evaluate publication bias. Heterogeneity was assessed using Cochran’s chi-squared test and quantified by I^2^ values. A random-effects model was applied during meta-analysis if heterogeneity was exhibited; otherwise, a fixed-effects model was used.

**Results:**

After reviewing 37,849 articles, 8 studies involving 3,324 housemaids were included for this systematic review and meta-analysis. The pooled prevalence of life time sexual violence among housemaids in Ethiopia was 46.26% (95% CI: 24.69, 67.84). The pooled prevalence was 55.43% (95% CI: 26.38, 84.47) for sexual harassment, 39.03% (95% CI: 14.55, 63.52) for attempted rape, and 18.85% (95% CI: 7.51, 30.19) for rape. Sexual violence is more likely among housemaid who previously lived rural residence (AOR = 2.25; 95% CI: 1.41, 3.60), drinks alcohol (AOR = 2.79 95% CI: 1.02, 4.56), and employer alcohol consumption (AOR = 6.01; 95% CI: 1.10, 32.96).

**Conclusion:**

This study revealed that the prevalence of sexual violence against housemaids in Ethiopia is high. Of the forms of sexual violence against housemaids, sexual harassment is high. Male employers are the vast majority of perpetrators of their housemaids. Thus, concerned stakeholders should develop and implement interventions that could empower housemaids in their struggle toward the elimination of sexual violence, create awareness for men, control and monitor the implementation of legislation and policies, and prompt punishment of the perpetrators.

*Systematic review and meta-analysis registration* PROSPERO CRD42021160511.

## Plain language summary

Sexual violence is the most common form of gender-based violence and has been a persistent problem in public health. Housemaids are the most vulnerable groups for any of the forms of sexual violence, as they face the greatest obstacles to gaining protection and necessary services. The sexual activity of domestic workers differs from that of the general population. Housemaids are more likely to be coerced into having sex and to have had sex before age 15 as compared to other young women. Housemaids often experience sexual violence by a person unknown to the victim, employers and male members of the household, brokers, or other intermediary persons. Sexual violence against housemaids remains usually concealed as victims cannot report such offenses. The most common reason for not reporting such violence was a lack of awareness of where to and for whom to report, a low level of education, and a fear of losing their work as they have few or no options for other work.

In Ethiopia, the national prevalence of sexual violence among housemaids is not investigated. Also, forms and determinants of sexual violence and identification of perpetrators have not been well described. Thus, a systematic review and meta-analysis was conducted to estimate the pooled prevalence of sexual violence amongst housemaids in Ethiopia. This systematic review and meta-analysis revealed that nearly half of housemaids experienced at least one form of sexual violence by different perpetrators. Of the forms of sexual violence against housemaids, sexual harassment is high. Male employers are the vast majority of perpetrators of their housemaids. Thus, concerned stakeholders should develop and implement interventions that could empower housemaids in their struggle toward the elimination of sexual violence, create awareness for men, and prompt punishment of the perpetrators.

## Background

Sexual violence is the most common form of gender-based violence, which is defined as any sexual act, attempt to obtain a sexual act, unwanted sexual comments or advances, acts to traffic, or other act directed against women’s sexuality using coercion, by any person regardless of their connection to the victim, in any setting, including but not limited to workplace and home [1, 2]. Sexual violence has many forms including sexual harassment, attempted rape, and complete rape [3, 4]. Minority and marginalized women are the most vulnerable groups to any of the forms of sexual violence, as they face the greatest obstacles to gaining protection and necessary services [5–7]. In a multiregional study conducted by World Health Organization (WHO), the prevalence of lifetime sexual violence among women was 30% [8]. Ethiopian Demographic and Health Survey (EDHS) 2016 reported that 10% of women aged 15–49 in Ethiopia have experienced sexual violence [9].

In developing countries, domestic work is an occupation for millions of women who represents 4–10% of the total workforce [10]. In Ethiopia, many women often migrate from rural to urban for domestic work [11, 12]. Domestic work puts housemaids in a highly disadvantaged position as it is one of the least protected sectors under labor law, in turn, worsens their vulnerability to sexual violence [13–16]. Most housemaids are obligated to attend their education on the night shift as they do not get the opportunity to continue their education on a regular program, which increases their vulnerability to sexual violence [6, 17, 18].

The sexual activity of domestic workers differs from that of the general population [19, 20]. Evidence revealed that housemaids were more likely to have been coerced into having sex and to have had sex before age 15 as compared to other young women [21, 22]. Housemaids often experience different types of sexual violence ranging from verbal harassment to completed rape by a person unknown to the victim, employers, male members of the household, brokers, or other intermediary persons [18, 23]. Sexual violence against housemaids remains usually concealed as victims cannot report such offenses. The most common reason for not reporting such violence were lack of awareness of where to and for whom to report, having a low level of education, and fear of losing their work as they have few or no options for other work [24–28].

Sexual violence is associated with reproductive health problems such as physical injury or death, sexually transmitted disease, unintended pregnancy, unsafe abortion, and risky sexual behavior. It has also a wide range of negative health outcomes such as depression, shame, humiliation, guilt, and post-traumatic stress disorders [29–33].

One of the key targets of the Sustainable Development Goals (SDG) is eliminating all forms of violence against women and girls [34]. Elimination of violence against women and girls is also essential to achieving most of the sustainable development goals predominantly Goal 3 and Goal 5 [34–36]. Attributable to this, several global and national organizations give due attention to the prevention of violence against women [3, 37, 38]. In Ethiopia, several policies and legislation have been also adopted and implemented to end violence against women [39–41].

In Ethiopia, extensive systematic reviews and meta-analyses have been conducted to determine the prevalence of sexual violence against female students [42, 43], and sexual violence in Ethiopian workplaces [44]. None of these systematic reviews have synthesized the results of prevalence studies conducted amongst housemaids. Except for individual studies with varying prevalence reports ranging from 12.3% [45] to 85.8% [46], there are no national pooled prevalence studies conducted on sexual violence among housemaids in Ethiopia. Also, forms and determinants of sexual violence and identification of perpetrators have not been well described. Hence, there was still a need for a systematic review and quantitative synthesis of prevalence studies conducted among housemaids. Thus, this systematic review and meta-analysis aimed to fill this gap by estimating the pooled prevalence and associated factors of sexual violence amongst housemaids in Ethiopia. Hereafter, understanding the magnitude of sexual violence against housemaids and its factors aims to contribute to design comprehensive interventions most fitted for domestic workers and inform policy makers.

## Methods

### Study design and reporting

A systematic review and meta-analysis was conducted to estimate the pooled prevalence and associated factors of sexual violence amongst housemaids in Ethiopia. This meta-analysis was carried out according to the Preferred Reporting Items for Systematic Reviews and Meta-Analyses (PRISMA) checklist [47]. The review was prospectively registered on the International Prospective Register of Systematic Reviews (PROSPERO) with the unique number CRD42021160511.

### Eligibility criteria

#### Study setting

Studies conducted only in Ethiopia were included.

#### Design

Observational studies reporting prevalence and/ or associated factors of sexual violence among housemaids were considered.

#### Publication status

Both published and unpublished studies were considered.

#### Language

The articles published and reported only in the English language were included. 

#### Publication year

All publications reported up to November 20, 2021, were considered.

#### Exposure

Predictors or determinants of sexual violence. The determinants are factors that increase or decrease the likelihood of sexual violence against housemaids.

#### Outcome

Lifetime prevalence of sexual violence against housemaids.

#### Exclusion criteria

Articles that were not fully accessible were excluded due to the inability to examine the quality of the articles without their full texts. Moreover, commentaries, case reports, case studies, qualitative studies, and review articles were excluded.

### Information sources and search strategy

A systematic and comprehensive search of the literature was conducted through the following databases: PubMed, Embase, Science Direct, HINARI, Scopus, Cochrane Library, and Google Scholar. The search was performed using the following key search terms: “Magnitude”, “Prevalence”, “sexual violence”, “sexual harassment”, “rape”, “sexual coercion”, "sex offense", "sexual abuse", “housemaid”, “domestic workers”, “associated factors”, “determinants”, “predictors” and “Ethiopia”. The search terms were used using different Boolean operators like “OR” or “AND”, and other truncations. In addition, the reference lists of all the included studies were also searched to identify any other studies that may have been missed by the search strategy. Moreover, the input of content experts and the Institutional Digital Library were searched to find unpublished articles.

### Study selection

All search records were exported to the EndNote X7.2.1 (Thomson Reuters, New York, USA) software citation manager to manage the duplication and screening process. Two (BDM and EBM) reviewers carefully read the titles and abstracts for the eligibility of the studies. Next, full-text articles were retrieved and evaluated to approve eligibility. The PRISMA flow diagram was used to summarize the overall study selection processes.

### Data extraction and quality assessment

Two reviewers (BDM and EBM) independently extracted relevant data from included articles using the Joanna Briggs Institute (JBI) tool for cross-sectional studies [48]. The following data were extracted from included articles: author, year of publication, study setting, study design, sample size, number of participants, response rate, lifetime sexual violence and the prevalence of different forms of sexual violence, perpetrators, and determinants of sexual violence.

Two reviewers independently assessed the methodological quality of all selected articles using the JBI critical appraisal checklist [49]. Any disagreements or unclear information were resolved through discussion. The overall quality of each study was rated from zero to ten-point scales. The study was scored ten if all of the quality measures were met whereas scored zero if none of the quality measures were met. The overall quality score of the study was based on the sum of points gained. Accordingly, primary studies scored ≥ 60% of the JBI criteria for evaluating the quality by two of the reviewers included in the meta-analysis [50].

### Operational definition

#### Sexual violence

A housemaid who experienced any sexual abuse, threatening, coercion, engaging in acts of sex without her will, or intimidated to have sex, and unwelcome jokes, verbal comments, touching, and kissing [51]. In this study, lifetime sexual violence against housemaids was considered if the original studies report any type or forms of sexual violence such as sexual harassment, attempted rape, or rape through working as a housemaid.

#### Sexual harassment

A housemaid who experienced unwanted sexual behaviors, such as unwelcome jokes and verbal comments, and unwelcome touching and kissing [52].

#### Rape

A housemaid who experienced any non-consensual penetration of the vagina, obtained by threatening or deception, by physical body harm, or without the housemaid’s consent [52].

#### Attempted rape

A housemaid who faced a trial to have sex without consent by threatening, or deception, by coercion, or when the housemaid cannot consent, without the actual penetration of the vagina [52].

### Data analysis

The selected studies and results were summarized using tables, figures, and forest plots. In a forest plot, the contribution of each study to the pooled estimates (weight) is represented by the area of a box. Statistical analysis was performed using STATA Version 14 statistical software. Heterogeneity between the included studies was assessed using Cochran’s chi-squared test and quantified by I^2^ values. The presence of heterogeneity across selected studies was assumed when p < 0.1 or I^2^ > 50% [53]. A random-effects model was applied to compute the pooled lifetime prevalence of sexual violence against housemaids if substantial heterogeneity was exhibited between studies; otherwise, a fixed-effects model was used.

Publication bias was evaluated by Egger’s rank test and Egger’s test for the presence of asymmetry funnel plot. Egger’s test was considered indicative of significant publication bias if a p-value ≤ 0.05 [54]. Sensitivity analysis was conducted to assess the presence and effect of outliers [55]. All statistical analyses were statistically significant at a level of 0.05.

## Results

### Study selection

**Fig. 1:**
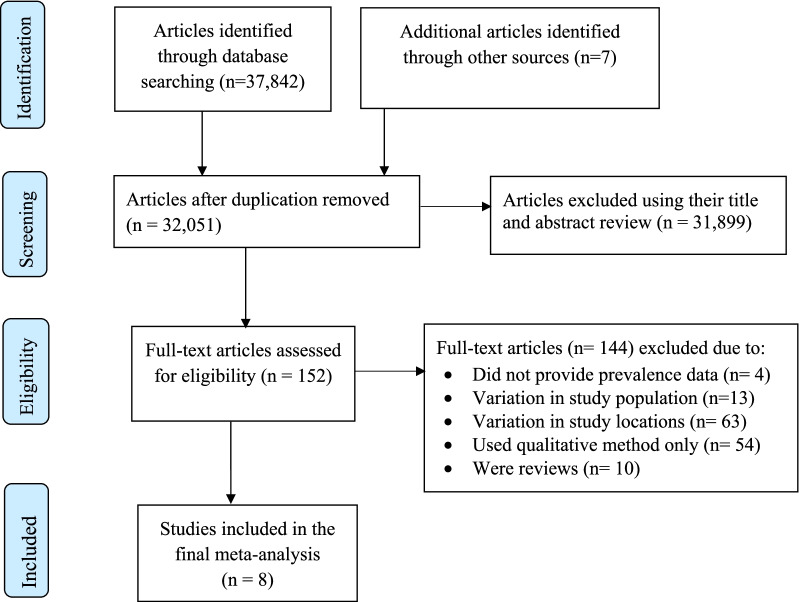
Flow diagram of the included studies in the meta-analysis of sexual violence against housemaids in Ethiopia

Initially, the literature search strategy generated 37,849 recorded articles. Then, 5,798 articles were removed due to duplication. Of the 32,051 remaining articles, 31,899 articles were excluded after reading their titles and abstracts. The remaining 152 potentially relevant full-text studies were independently evaluated based on the selection criteria. Further, 144 articles were excluded due to the following reasons: variation in the study population, variation in study locations; used qualitative method only; some were reviews; and not reporting the outcome of interest. Finally, 8 studies met the inclusion criteria and were included in the final meta-analysis (Fig. 1).

### Study characteristics

In this meta-analysis, a total of 3,324 housemaids were included from an estimated 3,443 participants, yielding a response rate of 96.5%. The sample size of the primary studies ranged from 75 to 826. The response rate ranges from 88.5% to 100%. All the selected articles used a cross-sectional study design. Concerning the study period, all the selected studies were conducted from 2006 to 2021. The highest prevalence of sexual violence against housemaids (85.8%) was found in a study done in Addis Ababa city [46] while the lowest prevalence (11.8%) was observed in a study done in Gondar city [56]. The eligible studies were from four geographical regions with four studies were conducted in Addis Ababa administrative city [45, 46, 57, 58], two studies in the Amhara region [56, 59], one study in Southern Nations, Nationalities, and Peoples’ Region (SNNPR) [60], and one was done in Harari region [61]. Quality assessment scores ranged from 6 to 9 points for each study. Two studies were scored 6 points, three were scored 7 points, two were scored 8 points, and one was scored 9 points (Table 1).

**Table 1:** Descriptive summary of studies included in the meta-analysis of sexual violence and associated factors among housemaids in Ethiopia, 2021

Author	Year	Region	Study area	Study design	Sample size	Response rate	Participants	Outcome (event)	Prevalence (%)	Quality score
Kefyalew et al. [59]	2019	Amhara	Debre Tabor	CBCS	636	100	636	177	27.8	8
Gezahegn et al. [60]	2021	SNNPR	Gedeo zone	CBCS	422	93.4	394	237	60.2	9
Alem et al. [45]	2019	Addis Ababa	Addis Ababa	CBCS	826	99.5	822	101	12.3	8
Yared et al. [57]	2006	Addis Ababa	Addis Ababa	CBCS	82	100	82	59	72	6
Yonas et al. [58]	2017	Addis Ababa	Addis Ababa	CBCS	545	96.1	524	155	29.6	7
Andualem et al. [61]	2014	Harari	Harar	CBCS	75	100	75	54	72	6
Muluken et al. [56]	2018	Amhara	Gondar city	CBCS	384	88.5	340	40	11.8	7
Mahilet et al. [46]	2015	Addis Ababa	Addis Ababa	CBCS	473	95.3	451	387	85.8	7

*CBCS* Community based cross-sectional

### Sensitivity analysis and publication bias

**Fig. 2:**
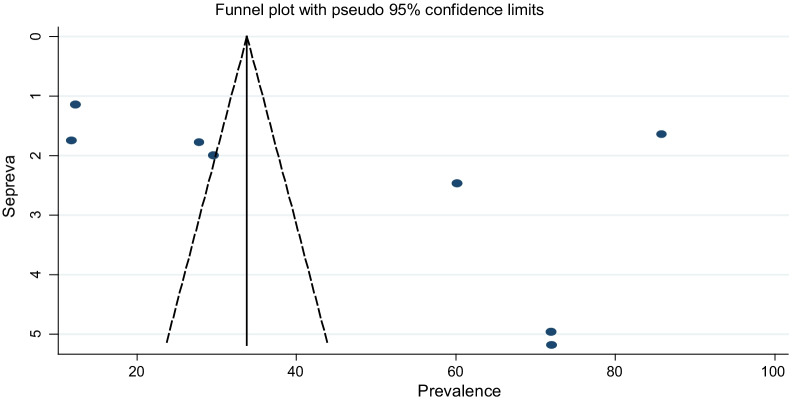
Graphic representation of publication bias using funnel plots of all included studies

Sensitivity analysis was conducted to assess the presence of individual studies that significantly influenced the pooled results. Accordingly, the result revealed that no single study significantly influenced the pooled prevalence of sexual violence against housemaids. Publication bias was evaluated by Egger’s rank test and Egger’s test for the presence of asymmetry funnel plot. The results of the tests revealed that publication bias was not observed, with the p-value for the Egger’s rank test being 0.275, and symmetry of the funnel plot (Fig. 2).

### Prevalence of sexual violence among housemaids

**Fig. 3:**
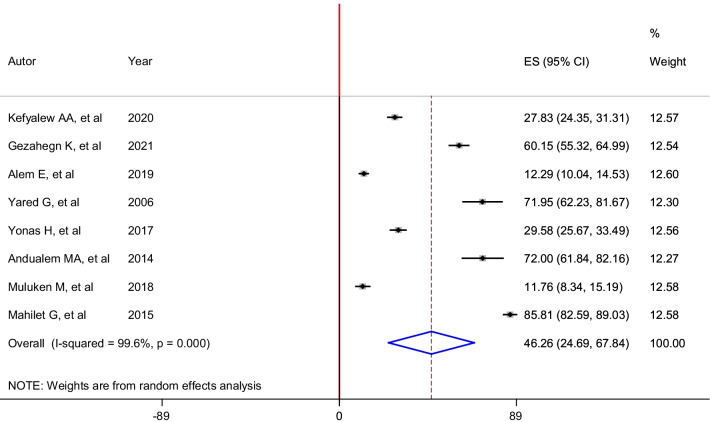
Forest plot of the pooled prevalence of sexual violence among housemaids in Ethiopia, 2021

The overall pooled prevalence of lifetime sexual violence among housemaids in Ethiopia was 46.26% (95% CI: 24.69, 67.84). The random-effects model was performed as the analysis revealed significant heterogeneity (I^2^ = 99.6%, p < 0.01) between the included studies (Fig. 3).

### Types of sexual violence

**Fig. 4:**
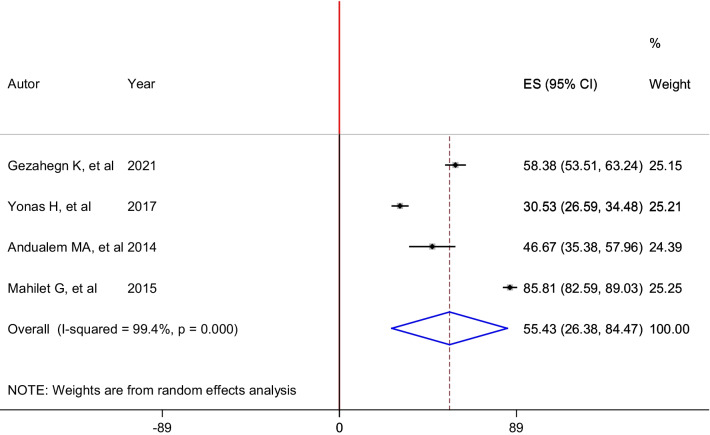
Forest plot of the pooled prevalence of sexual harassment among housemaids in Ethiopia, 2021

In this review, the prevalence of the different types of sexual violence was assessed. Accordingly, sexual harassment was the most prevalent type of sexual violence, with a pooled prevalence of 55.43% (95% CI: 26.38, 84.47). In this analysis, a random-effects model was performed as high heterogeneity (I^2^ = 99.4%, p < 0.01) was exhibited across the included six studies (Fig. 4).

**Fig. 5:**
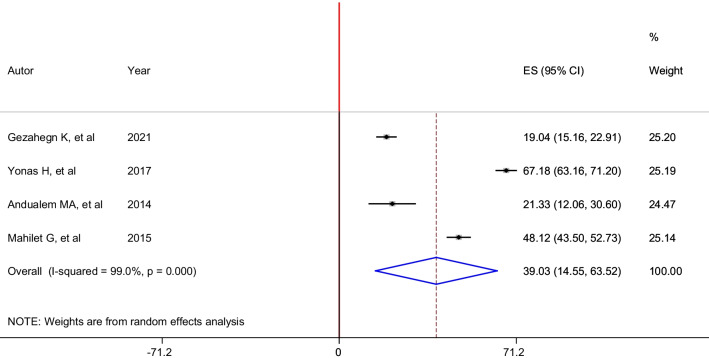
Forest plot of the pooled prevalence of attempted rape among housemaids in Ethiopia, 2021

Four studies reported the prevalence of attempted rape. Hence, the pooled prevalence of lifetime attempted rape among housemaids in Ethiopia was 39.03% (95% CI: 14.55, 63.52). In this analysis, a random-effects model was performed as significant heterogeneity (I^2^ = 99.0%, p < 0.01) was exhibited across the included studies (Fig. 5).

**Fig. 6:**
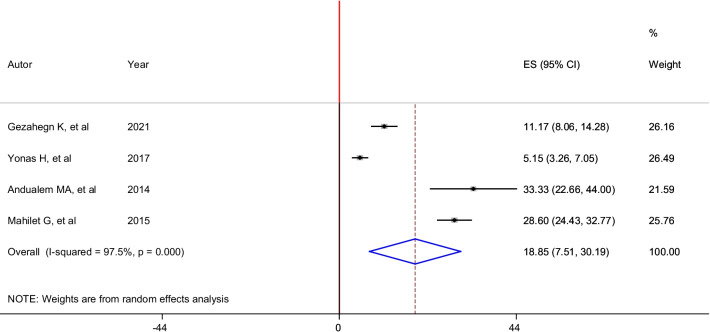
Forest plot of the pooled prevalence of completed rape among housemaids in Ethiopia, 2021

According to the reports of six studies, the pooled prevalence of lifetime completed rape among housemaids in Ethiopia was 18.85% (95% CI: 7.51, 30.19). In this analysis, a random-effects model was performed as significant heterogeneity (I^2^ = 97.5%, p < 0.01) was exhibited across the included studies (Fig. 6).

### Perpetrators of sexual violence

In this review, the perpetrators of sexual violence against housemaids were identified. The prevalence of each perpetrator reported in more than one primary study was pooled quantitatively. Hence, the main perpetrators of sexual violence against housemaids were male employers, with a pooled prevalence of 45.46% (95% CI: 27.75, 63.17) followed by brokers or other intermediary persons 28.0% (95% CI: 11.36, 44.64) (Table 2).

**Table 2:** Perpetrators of sexual violence against housemaids in Ethiopia, 2021

Perpetrators of sexual violence	Studies	Estimates (95% CI)	Pooled prevalence % (95% CI)	Test of heterogeneity		
				I^2^	Q	P
Male employer	Yared et al. 2006 [57]	21.95 (12.99, 30.91)	45.46 (27.75, 63.17)	97.1%	5.03	< 0.001
	Yonas et al. 2017 [58]	51.91 (47.63, 56.19)				
	Andualem et al. 2014 [61]	76.00 (66.33, 85.67)				
	Mahilet et al. 2015 [46]	32.59 (28.27, 36.92)				
Broker or other intermediary person	Gezahegn et al. 2021 [60]	22.84 (18.70, 26.99)	28.00(11.36, 44.64)	97.7%	3.30	< 0.001
	Yared et al. 2006 [57]	69.51 (59.55, 79.48)				
	Andualem et al. 2014 [61]	10.67 (3.68, 17.65)				
	Mahilet et al. 2015 [46]	11.53 (8.58, 14.48)				
Male members of employer household	Yared et al. 2006 [57]	30.49 (20.52, 40.45)	26.52 (23.01, 30.03)	0.0%	14.8	0.404
	Yonas et al. 2017 [58]	25.95 (23.10, 30.03)				
Unknown by victim	Gezahegn et al. 2021 [60]	31.73 (27.13, 36.32)	25.77 (15.32, 36.21)	95.2%	4.84	< 0.001
	Yared et al. 2006 [57]	9.76 (3.33, 16.18)				
	Yonas et al. 2017 [58]	38.17 (34.01, 42.33)				
	Andualem et al. 2014 [61]	12.00 (4.65, 19.35)				
	Mahilet et al. 2015 [46]	35.70 (31.28, 40.12)				

### Factors associated with sexual violence among housemaids

In this review, some of the factors associated with sexual violence against housemaids were pooled quantitatively and some were not because of inconsistent classification (grouping) of the independent variables to the outcome variable. Thus, those determinants consistently reported in more than one original study were included in this meta-analysis.

Two studies indicated that housemaids who came from rural areas were more likely to experience sexual violence. The pooled odds ratio indicated that housemaids who previously lived in rural areas were 2.25 times (AOR = 2.25; 95% CI: 1.41, 3.60) more likely to experience sexual violence as compared to those from urban areas. Two studies also indicated that housemaids who drink alcohol were more likely to experience sexual violence. The overall estimates revealed that housemaids who drink alcohol were 2.79 times (AOR = 2.79 95% CI: 1.02, 4.56) more likely to experience sexual violence as compared to their counterparts, those who do not drink alcohol. Furthermore, employer alcohol consumption was another significant factor associated with sexual violence against housemaids. Housemaids whose male employer drinks alcohol were 6.01 times (AOR = 6.01; 95% CI: 1.10, 32.96) more likely to experience sexual violence compared to those whose employers do not drink alcohol (Table 3).

**Table 3:** Factors associated with sexual violence against housemaids in Ethiopia, 2021

Variables	Studies	Odds ratio (95% CI)	Pooled odds ratio (95% CI)	Test of heterogeneity		
				I^2^	Q	P
Previous rural residence	Kefyalew et al. 2019 [59]	2.73 (1.31, 5.69)	2.25 (1.41, 3.60)	0.0%	3.39	0.503
	Gezahegn et al. 2021 [60]	1.97 (1.07, 3.63)				
Housemaid alcohol consumption	Gezahegn et al. 2021 [60]	1.91 (0.97, 2.85)	2.79 (1.02, 4.56)	83.4%	3.09	0.014
	Mahilet et al. 2015 [46]	3.72 (2.62, 4.82)				
Male employers alcohol consumption	Kefyalew et al. 2019 [59]	2.56 (1.61, 4.07)	6.01 (1.10, 32.96)	94.6%	2.07	< 0.001
	Mahilet et al. 2015 [46]	14.5 (7.63, 27.7)				

## Discussion

This meta-analysis revealed that the pooled prevalence of lifetime sexual violence among housemaids in Ethiopia was 46.26%. Even though there was no similar meta-analysis conducted on this specific research question, the finding was higher than the prevalence of workplace sexual violence in Ethiopia reported as 22% [44]. However, the finding was lower than workplace sexual violence among Nigerian employees reported as 63.8% [62]. The possible reason could be attributed to the difference in workplaces, population, and practice of prohibited workplace sexual violence legislation. The studies mentioned above were conducted on a different group of the population including government employees and students, who could have better access to information regarding the violence that could help to easily protect themselves. Furthermore, the variation may be due to differences in accessibility of information on consequences, prevention and control of sexual violence.

The results of this review revealed that sexual harassment (55.43%) was the most prevalent type of sexual violence, followed by attempted rape (39.03%), and completed rape (18.85%) among housemaids in Ethiopia. The difference in the pooled prevalence estimates of sexual violence types was also observed in a systematic review and meta-analysis of workplace sexual violence conducted in Ethiopia [44]. This finding implies the necessity for comprehensive legislation and prohibitions of violence to address all the forms of sexual violence occurring among the Ethiopian domestic workers. This finding also infers the need that all employers to implement the labor proclamation set by the ministry of labor and social affairs of Ethiopia. The extent of diverse types of sexual violence among housemaids indicates a need for better protection and broad action to create a safe working environment and to end violence against women. Evidence indicates diverse types of sexual violence remain a significant obstacle to the achievement of the 2030 agenda for sustainable development goals, and women’s and girls’ human rights [63].

In this review, the main perpetrators of sexual violence against housemaids were male employers, brokers or other intermediary people, a male member of the household, unknown person by the victim. This finding indicates that perpetrators of housemaids are more likely to commit the assault at the employer’s home, both outdoors and indoors. Literature showed that perpetrators of sexual violence are more likely to commit the attack outdoors, and indoors, most commonly in the victim’s or perpetrator’s home [8, 64]. This finding could also indicate that perpetrators might consciously plan to commit sexual assault because men employers are responsible for the vast majority of sexual violence against housemaids.

This review identified that housemaids who previously lived in rural areas were more likely to experience sexual violence by different perpetrators. This finding is supported by reviews conducted in Ethiopia [43] and Sub-Saharan Africa [65]. This might be explained by the fact that housemaids from the rural areas have a low level of awareness of sexual violence which increases the possibility of being victimized by perpetrators. There are some literature indicating that sexual violence is more likely among women in rural areas due to harmful beliefs and traditions, low level of awareness of sexual violence, less exposure to sexual and reproductive health information, and inadequate as well as the inaccessibility to legal services [66–68].

This review also indicated that housemaids who drink alcohol were more likely to experience sexual violence. The result is supported by other systematic reviews [43, 65, 69]. This implies that alcohol use could diminish women’s problem-solving skills and ability to anticipate the potential risks of sexual violence. This might be also explained by alcohol use increasing individuals’ willingness to take risks, and making them weak to protect themselves [70, 71]. The literature revealed that individuals who consume alcohol or substances couldn’t interpret and effectively respond to the warning signs of sexual assault [72, 73].

Furthermore, employer alcohol consumption was another significant factor associated with sexual violence against housemaids. Housemaids whose male employer drinks alcohol were more likely to experience sexual violence. This could be attributed to the detrimental influence of alcohol on the level of risk identification and decision-making skill. In addition, this could relate to the depressive and mental impairment effect of alcohol, which encourages employers to commit violence against their housemaids [74].

This systematic review and meta-analysis have the following potential limitations: Though heterogeneity between studies was exhibited, the important sources of heterogeneity were not addressed. In addition, some of the selected studies had a small sample size which may influence the estimated reports. Besides, the causal relationship between outcome and determinants may not be established as all the included studies were cross-sectional. Moreover, the selected studies represented only four geographical regions, which might affect the estimated prevalence. Nevertheless, this study provided the first quantitative pooled prevalence of sexual violence against housemaids in Ethiopia, to the best of our knowledge.

This systematic review and meta-analysis revealed that nearly half of housemaids experienced sexual violence by different perpetrators. This finding implies the necessity of strengthening regulations or policy interventions specific to violence against housemaids by considering them as they are underprivileged group. The finding also infers the need of better enactment for awareness creation on the concept of violence against housemaids for the society. Moreover, the finding may be attributed to the role of understanding the burden of sexual violence for the possibility and effectiveness of prevention and control interventions. Evidence showed that understanding the burden of sexual violence would help for the possibility and effectiveness of prevention and control programs [75, 76].

This review provides vital evidence to inform policy-makers, health programmers, and other relevant stakeholders to prevent and control sexual violence against housemaids. Some factors were identified that are associated with increased experiences of sexual violence among housemaids. Hence, prioritizing the factors and the prevention of sexual violence should be commenced sooner rather than later. A variety of forms of sexual violence were identified that are commonly experienced against housemaids. Thus, information provision, education, and training programs are necessary to assist and empower housemaids. Perpetrators of sexual violence were identified that are commonly committing sexual assault against housemaids. Hence, enforcing appropriate legislation and policies such as encouraging housemaids to report such acts and prompt punishment of the perpetrators, and public awareness creation campaigns are crucial to end sexual violence.

## Conclusions

This study revealed that the prevalence of sexual violence against housemaids in Ethiopia is high. Of the forms of sexual violence against housemaids, sexual harassment is high. Male employers are the vast majority of perpetrators of their housemaids. Predictors that increase experiences of sexual violence against housemaids were previous rural residence, housemaid alcohol use, and employer alcohol consumption. Thus, policy-makers, health programmers, and other relevant stakeholders should develop and implement interventions that could empower housemaids in their struggle toward the elimination of sexual violence, create awareness for men, control and monitor the implementation of legislation and policies, and prompt punishment of the perpetrators. Moreover, primary preventative methods of sexual violence should incorporate interventions that target housemaids, and increase access to sexual and reproductive health information.

## Data Availability

All relevant data is included within the manuscript file.

## Abbreviations

AOR: Adjusted odds ratio
CBCS: Community based cross-sectional
CI: Confidence intervals
JBI: Joanna Briggs Institute
OR: Odds ratio
